# Variability in uptake efficiency for pulsed versus constant concentration delivery of inhaled nitric oxide

**DOI:** 10.1186/2045-9912-4-1

**Published:** 2014-01-22

**Authors:** Andrew R Martin, Chris Jackson, Ira M Katz, Georges Caillibotte

**Affiliations:** 1 Delaware Research and Technology Center, American Air Liquide, Newark, DE, USA; 2 Medical Gases Group, Air Liquide Santé International, 1 chemin de la Porte des Loges, Les Loges-en-Josas 78354, France; 3 Department of Mechanical Engineering, Lafayette College, Easton, PA, USA; 4 Current address: Department of Mechanical Engineering, University of Alberta, Edmonton, AB, Canada; 5 Current address: 99 Fieldsend, Beaconsfield, QC H9W 5J2, Canada

**Keywords:** Lung model, Trumpet model, Medical gas, Nitric oxide, Gas transport, Conducting airways, Mixing, Dispersion, Bolus, Pulse

## Abstract

**Background:**

Nitric oxide (NO) is currently administered using devices that maintain constant inspired NO concentrations. Alternatively, devices that deliver a pulse of NO during the early phase of inspiration may have use in optimizing NO dosing efficiency and in extending application of NO to long-term use by ambulatory, spontaneously breathing patients. The extent to which the amount of NO delivered for a given pulse sequence determines alveolar concentrations and uptake, and the extent to which this relationship varies with breathing pattern, physiological, and pathophysiological parameters, warrants investigation.

**Methods:**

A mathematical model was used to analyze inhaled nitric oxide (NO) transport through the conducting airways, and to predict uptake from the alveolar region of the lung. Pulsed delivery was compared with delivery of a constant concentration of NO in the inhaled gas.

**Results:**

Pulsed delivery was predicted to offer significant improvement in uptake efficiency compared with constant concentration delivery. Uptake from the alveolar region depended on pulse timing, tidal volume, respiratory rate, lung and dead space volume, and the diffusing capacity of the lung for NO (D_L_NO). It was predicted that variation in uptake efficiency with breathing pattern can be limited using a pulse time of less than 100 ms, with a delay of less than 50 ms between the onset of inhalation and pulse delivery. Nonlinear variation in uptake efficiency with D_L_NO was predicted, with uptake efficiency falling off sharply as D_L_NO decreased below ~50-60 ml/min/mm Hg. Gas mixing in the conducting airways played an important role in determining uptake, such that consideration of bulk convection alone would lead to errors in assessing efficiency of pulsed delivery systems.

**Conclusions:**

Pulsed NO delivery improves uptake efficiency compared with constant concentration delivery. Optimization of pulse timing is critical in limiting intra- and inter-subject variability in dosing.

## Background

Inhaled nitric oxide (NO) has been shown to act as a selective pulmonary vasodilator [1,2]. Since this discovery more than 20 years ago, use of NO has become widespread for treating persistent pulmonary hypertension of the newborn (PPHN) [3], improving oxygenation in adult patients with acute lung injury or the acute respiratory distress syndrome [4], and in alleviating pulmonary hypertension in adults and children following cardiac surgery [5,6]. These settings generally require NO to be delivered to mechanically ventilated patients. To do so, delivery devices have been developed which administer NO from a source cylinder containing 100–1000 ppm NO (800 ppm NO in North America) in balance nitrogen, to the inspiratory limb of the patient breathing circuit. The flow rate of NO injection is adjusted in proportion to the flow of air/oxygen in the circuit so as to produce a constant NO concentration in the inspired gas [7,8]. Accordingly, dosing recommendations for inhaled NO have been established based on the NO concentration in inspired gas [4,9].

For some time, researchers have speculated that the dose–response relationship for inhaled NO might be better described in terms of alveolar concentration or uptake from the lungs, rather than inhaled concentration [10,11]. Such considerations have often been made in the context of pulsed NO delivery systems, which deliver a predetermined volume of NO in the early portion of inhalation, with the goal of maximizing delivery to the peripheral, gas exchange lung regions, while avoiding delivery of NO to the anatomical dead space near the end of inhalation [11-15]. Pulsed systems may have use in optimizing dosing and minimizing total NO usage for intubated, ventilated patients, but also in extending application of NO to long-term use by ambulatory, spontaneously breathing patients. However, these systems are inherently poorly described using the established dose metric of inhaled NO concentration, as the concentration is intentionally varied over each inhalation. Instead, they are potentially well adapted for dosing in terms of the absolute amount (volume, mass, moles) of NO delivered per breath or over a given time period. The extent to which the amount of NO delivered for a given pulse sequence determines alveolar concentrations and uptake, and the extent to which this relationship varies with breathing pattern, and with physiological and pathophysiological parameters, is presently unclear, and thus warrants study.

This article describes a mathematical model developed to couple gas mixing and transport through the conducting airways with uptake from the alveolar lung region. Continuous or pulsed NO delivered to the extrathoracic airway was transported through serial airway generations by a combination of bulk convection and axial dispersion. Uptake from the alveolar region was determined as a function of alveolar volume and NO concentration, as well as the diffusing capacity of the lung for NO. The model was used to evaluate equivalency, in terms of alveolar concentration and uptake, between constant concentration and pulsed NO dosing, and to establish framework recommendations on pulse volume, duration, and delay time (between onset of inhalation and pulse delivery) for optimal uptake efficiency. In addition, the significance of axial dispersion on NO transport, and ultimately uptake, was evaluated, as was the sensitivity of uptake efficiency to the lung’s diffusing capacity for NO.

## Methods

### Modeling approach

A single compartment, single path lung model was employed. The lung was divided into a gas-conducting region and an alveolar region. It was assumed that zero uptake of NO occurred across the conducting airways, whereas uptake from the alveolar region into the capillaries was modeled with a single transfer factor specifying volume uptake per unit time per unit partial pressure (i.e., D_L_NO – the diffusing capacity of the lung for NO). Inputs to the model included the D_L_NO, along with the functional residual capacity (FRC) of the lung, the dimensions of the conducting airways, the breathing pattern (inhaled and exhaled volumetric flow rates vs. time), and the variation in inhaled NO concentration specified over time. Outputs of the model included the time-varying concentrations of NO in each generation of the conducting airways and in the alveolar region, the rate at which NO was taken up into the capillaries, and the NO concentration in the exhaled gas.

### Conducting airways

The tracheobronchial airways making up the gas-conducting region of the lung can be described as a symmetric, bifurcating network of rigid, cylindrical tubes starting at the trachea and extending to the terminal bronchioles. In the present work, baseline dimensions of the conducting airways were specified according to the adult model provided by Finlay *et al.*[16], based on airway data from Phillips *et al*. [17]. These are provided in Table 1, and correspond to a FRC of the lung of 3000 ml. Airway lengths and diameters were then scaled with FRC according to

(1) ℓ=ℓ0FRC3000ml13

where ℓ represents the scaled length or diameter, and ℓ_o_ represents the corresponding length or diameter at FRC of 3000 ml.

**Table 1:** **Conducting airway dimensions from Finlay et al.**[16]

**GEN#**	**Diameter (cm)**	**Length (cm)**	Volume* (ml)
0	1.80	12.46	32.05
1	1.41	3.61	11.35
2	1.12	2.86	11.17
3	0.89	2.28	11.22
4	0.71	1.78	11.13
5	0.57	1.13	9.03
6	0.45	0.90	9.29
7	0.36	0.83	11.00
8	0.29	0.75	12.22
9	0.22	0.65	12.46
10	0.16	0.56	11.79
11	0.12	0.45	10.67
12	0.09	0.36	9.74
13	0.07	0.28	9.53
14	0.06	0.22	10.49

*Total volume of 2^*n*^ airways in generation *n*.

Proximal to the trachea, an extrathoracic airway was defined by a characteristic diameter and length of 0.75 cm and 30 cm, respectively, to represent an adult endotracheal tube.

Dispersion of inhaled NO through the extrathoracic and conducting airways was analyzed following an approach analogous to the Eulerian dynamical framework outlined by Finlay [18] for modeling aerosol transport through the respiratory tract. In brief, the convection-diffusion equation was solved in one dimension over a single path, with the coordinate *x* representing the depth into the lung along that path. The conducting airway dimensions described above were used to define the total cross-sectional area of airways in the lung as a function of *x*. It was assumed that the flux of NO through airway walls was zero, and that conducting airway cross-sections remained constant over time. The 1D transport equation could then be written in differential form as

(2) $$\frac{\partial }{\partial t}\left(A\bar{c}\right)+\frac{\partial }{\partial x}\left(A\bar{c}\bar{u}\right)=\frac{\partial }{\partial x}\left(AD_{m}\frac{\partial \bar{c}}{\partial x}\right)$$

where *A* is the total cross-sectional area of airways in the lung at a given depth *x*, $\bar{c}$ is the NO concentration averaged over the area *A*, $\bar{u}$ is the average flow velocity in the *x*-direction over the area *A*, and *D*_*m*_ is the molecular diffusivity of NO in air*.*

Equation (2) assumes both the velocity and the NO concentration are uniform across the area *A*. In general, these variables are not uniform, and their variation across the entire airway cross section at a given lung depth is complex. To preserve the simplicity of the 1D model, a common approach is to replace *D*_*m*_ with an effective diffusion coefficient *D*_*eff*_, the latter acting as a catch-all term encompassing both molecular diffusion and various convective-diffusive dispersion phenomena arising from non-uniform velocity and concentration profiles. The effective diffusion coefficient can thus be written as the summation

(3) $$D_{\mathrm{eff}}=D_{m}+D_{c-d}$$

where *D*_*c-d*_ represents the contribution of convective-diffusive dispersion to the total effective diffusive flux.

Ben-Jebria [19] presented compelling arguments that flow patterns through the conducting airways are sufficiently complex that gas mixing cannot be described using the laminar dispersion model described by Taylor [20], but should instead be estimated based on experiments performed by Scherer *et al.*[21] on gas dispersion in a physical model of the first five branches of the conducting airways. In the latter approach, the dispersion coefficient is

(4) $$D_{c-d}=\alpha \bar{u}d$$

where α is an empirical constant equal to 1.08 during inhalation and 0.37 during exhalation, and *d* is the diameter of the airway.

**Figure 1:**
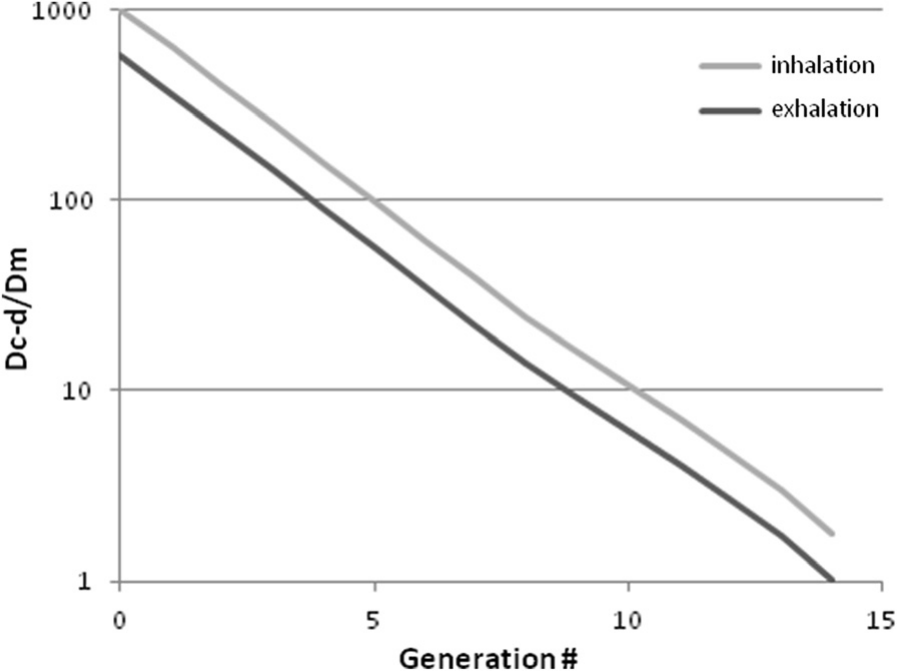
Ratio between the two components of the effective diffusion coefficient during inhalation and exhalation over the conducting airways.

Using equation (4), *D*_*c-d*_ may be calculated and compared to *D*_*m*_ for each generation of the conducting airways. This was done for airway velocities *ū* corresponding to a 300 ml/s inspiratory flow rate and 500 ml/s expiratory flow rate using airway diameters specified in the Finlay *et al.*[16] airway model described above, and using a molecular diffusivity of NO in air of 0.23 cm^2^/s. The comparison is presented in Figure 1, where it can be seen that *D*_*c-d*_ is much greater than *D*_*m*_ during both inhalation and exhalation through most of the conducting airways, but that molecular diffusivity becomes non-negligible in the small airways approaching the transition to the alveolar region.

With *D*_*m*_ replaced by *D*_*eff*_, equation (2) can be reorganized as

(5) $$A\frac{\partial \bar{c}}{\partial t}=\frac{\partial }{\partial x}\left(AD_{\mathrm{eff}}\frac{\partial \bar{c}}{\partial x}\right)-\frac{\partial }{\partial x}\left(A\bar{c}\bar{u}\right)$$

For each case studied, the time variation of NO concentration was specified at the entrance to the extrathoracic airway, the concentration gradients at the entrance and at the boundary between conducting airways and the alveolar region were set to zero, and the initial NO concentration was set to zero throughout the geometry. That is

(6) $$\begin{array}{l} \;\bar{c}\left(x=0,t\right)=f\left(t\right)\\ {{\left(\frac{\partial \bar{c}}{\partial x}\right|}^{t}}_{x=0}={{\left(\frac{\partial \bar{c}}{\partial x}\right|}^{t}}_{x=L}=0\\ \;\bar{c}\left(x,\;t=0\right)=0 \end{array}$$

where *f(t)* specifies the inhaled NO concentration over time, and *x = L* denotes the boundary between conducting airways and alveolar region.

### Alveolar region

The alveolar region was modeled as a single, well-mixed compartment that expanded and contracted during the breathing cycle consistent with the inhaled and exhaled tidal volume. The mass conservation of NO in the alveolar region during inhalation can be written as

(7) $$\frac{d\left(c_{\mathrm{alv}}V_{\mathrm{alv}}\right)}{dt}={\bar{c}}_{{}_{14}}Q-c_{\mathrm{alv}}D_{L}\mathrm{NO}$$

and during exhalation as

(8) $$\frac{d\left(c_{\mathrm{alv}}V_{\mathrm{alv}}\right)}{dt}=c_{\mathrm{alv}}Q-c_{\mathrm{alv}}D_{L}\mathrm{NO}$$

where

(9) $$\frac{dV_{\mathrm{alv}}}{dt}=Q\left(t\right)$$

and where *Q* is the instantaneous volumetric flow rate of gas into or out of the lungs, *V*_*alv*_ is the volume of the alveolar region, *c*_*alv*_ and ${\bar{c}}_{14}$ represent the NO concentrations in the alveolar region and in the terminal bronchioles (generation 14), respectively, and *D*_*L*_*NO* is the diffusing capacity of the lung for NO. D_L_NO is normally specified as the volume uptake of NO per unit time per unit partial pressure, but as written in equations (7) and (8) D_L_NO must be expressed in units of the quantity of NO conserved (i.e., mass, moles, or volume at STP) per unit time per unit concentration.

After expanding the left hand sides of equations (7) and (8) using the product rule, and rearranging terms, the rate of change of alveolar concentration of NO was determined during inhalation as

(10) $$\frac{dc_{\mathrm{alv}}}{dt}=\frac{\left({\bar{c}}_{{}_{14}}-c_{\mathrm{alv}}\right)Q-c_{\mathrm{alv}}D_{L}\mathrm{NO}}{V_{\mathrm{alv}}}$$

and during exhalation as

(11) $$\frac{dc_{\mathrm{alv}}}{dt}=\frac{-c_{\mathrm{alv}}D_{L}\mathrm{NO}}{V_{\mathrm{alv}}}$$

### Numerical procedure

Equation (5) was discretized and solved through the extrathoracic and conducting airways over finite divisions of length using an upwind approximation for the convective term and a central difference approximation for the diffusive term. The discretized transport equation during inhalation was

(12) $$\begin{array}{l} \;C_{i}^{t}=C_{i}^{t-\mathrm{\Delta t}}+\left(C_{i+1}^{t-\mathrm{\Delta t}}-C_{i}^{t-\mathrm{\Delta t}}\right)\frac{{\bar{u}}_{i}^{t}}{l_{i}}\mathrm{\Delta t}\\ +\frac{1}{2}\left[\left(A_{i-1}+A_{i}\right)\left(\mathrm{Def}f_{i-1}^{t}+\mathrm{Def}f_{i}^{t}\right)\left(\frac{C_{i-1}^{t-\mathrm{\Delta t}}-C_{i}^{t-\mathrm{\Delta t}}}{l_{i-1}+l_{i}}\right)\right)\\ \left(\;-\left(A_{i}+A_{i+1}\right)\left(\mathrm{Def}f_{i}^{t}+\mathrm{Def}f_{i+1}^{t}\right)\left(\frac{C_{i+}^{t-\mathrm{\Delta t}}}{l_{i}+l_{i+1}}\right)\right]\frac{\mathrm{\Delta t}}{l_{i}A_{i}} \end{array}$$

and during exhalation was

(13) $$\begin{array}{l} \;C_{i}^{t}=C_{i}^{t-\mathrm{\Delta t}}+\left(C_{i-1}^{t-\mathrm{\Delta t}}-C_{i}^{t-\mathrm{\Delta t}}\right)\frac{{\bar{u}}_{i}^{t}}{l_{i}}\mathrm{\Delta t}\\ +\frac{1}{2}\left[\left(A_{i-1}+A_{i}\right)\left(\mathrm{Def}f_{i-1}^{t}+\mathrm{Def}f_{i}^{t}\right)\left(\frac{C_{i-1}^{t-\mathrm{\Delta t}}-C_{i}^{t-\mathrm{\Delta t}}}{l_{i-1}+l_{i}}\right)\right)\\ \left(\;-\left(A_{i}+A_{i+1}\right)\left(\mathrm{Def}f_{i}^{t}+\mathrm{Def}f_{i+1}^{t}\right)\left(\frac{C_{l_{i}}^{t-\mathrm{\Delta t}}-C_{i+1}^{t-\mathrm{\Delta t}}}{l_{i}+l_{i+1}}\right)\right]\frac{\mathrm{\Delta t}}{l_{i}A_{i}} \end{array}$$

where *C*_*i*_^*t*^ is the NO concentration in the i^th^ division at time t, and *l*_*i*_ is the length of the i^th^ division. The parameters ${\bar{u}}_{i}^{t}$ and *D*_*eff,i*_^*t*^ were known, and prescribed at the start of the procedure based on the specified breathing pattern and airway dimensions.

Alveolar concentrations were calculated directly using the finite difference versions of equations (10) and (11) during inhalation and exhalation, respectively.

Concentrations throughout the respiratory tract were calculated explicitly over time via the Euler method. To assess time step and spatial grid size dependence, initial calculations were performed with 1000, 2500, 5000, or 10000 equal time steps per breath, and for 1, 2, 3, or 5 equal length divisions per generation. For constant inhaled NO concentrations, increasing the number of time steps per breath or divisions per generation had negligible effect on NO uptake and uptake efficiency, save that in the case of 5 divisions per generation 5000 time steps per breath were required for convergence. For calculations performed for pulsed NO delivery, increasing the number of divisions per generation from 1 to 3 reduced NO uptake and uptake efficiency slightly, while further increase above 3 divisions had negligible effect. Effects of time step size were negligible, save that again 5000 time steps per breath were required for convergence in the case of 5 divisions per generation. For all combinations of time step and divisions, convergence or divergence of the solution was well predicted by applying the practical criterion given by Darquenne and Paiva [22] at the terminal conducting airway (generation 14 in the present model). Results presented below were obtained using 5 divisions per generation and 5000 time steps per breath.

### Cases studied

**Figure 2:**
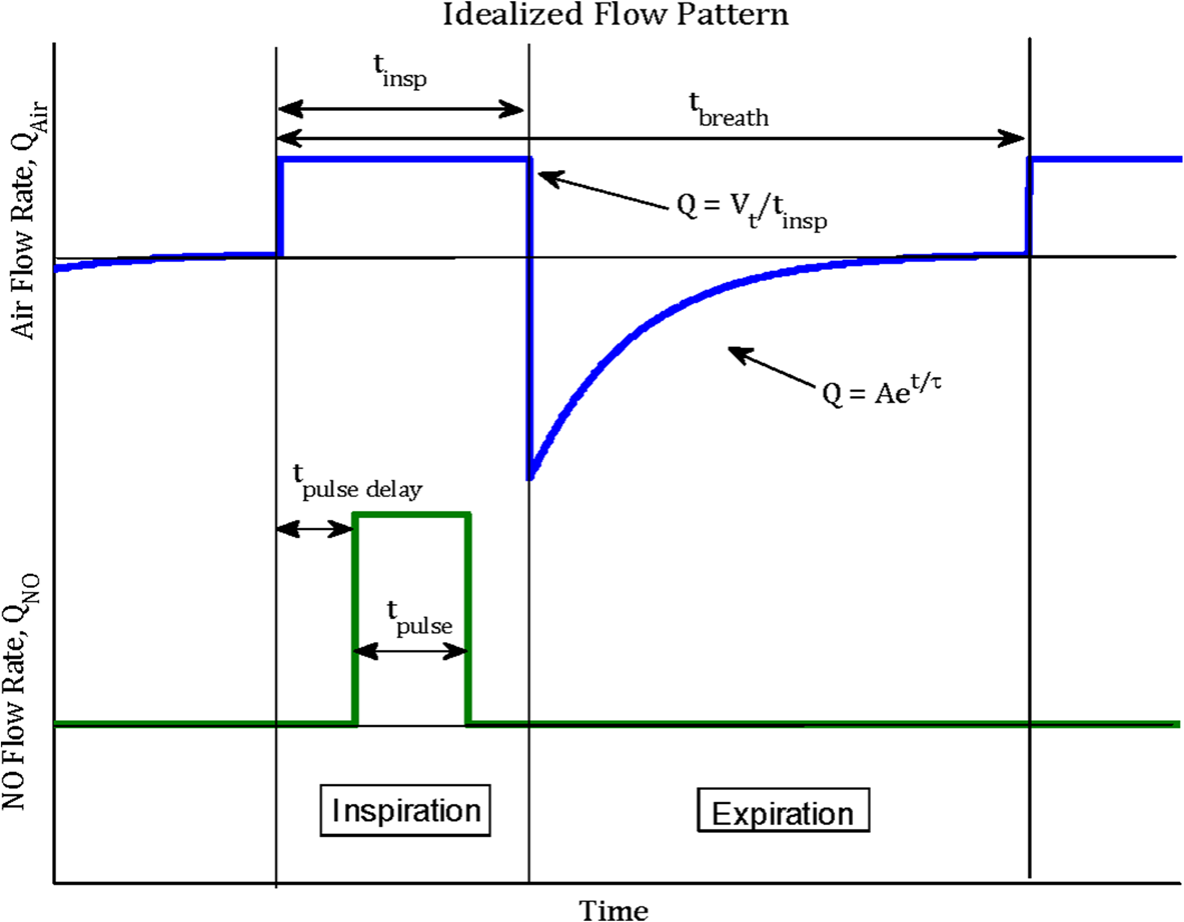
Idealized breathing pattern (top) and pulse form (bottom) used as inputs to the model.

The model presented above was first verified through comparison with a model previously proposed by Heinonen *et al.*[11], which compared well with *in vivo* exhaled NO concentrations measured by the same authors during continuous and pulsed delivery of NO to intubated, mechanically ventilated pigs. For the purpose of comparison, all parameters were set equal to those reported by Heinonen *et al.*[11]. The idealized flow pattern described in Figure 2 was adopted, with tidal volume of 400 ml, respiratory rate of 20 min^-1^, inspiratory time fraction equal to 1/3 of the breath, and the expiratory time constant equal to 0.37 s. In addition, the D_L_NO was set to 48 ml/min/mm Hg, FRC to 700 ml, and airway dimensions were scaled to yield an anatomical dead volume of 150 ml. Following Heinonen *et al.*[11], the NO concentration in inhaled gas was either set to a constant 5 ppm throughout inhalation, or, for the case of pulsed delivery of 100 nmol NO/min, a 110 μl pulse containing 1000 ppm NO in balance nitrogen was injected during only the first third of each inhalation at a flow rate of 0.33 ml/s. In the latter case, the NO/N_2_ stream was assumed to be well mixed with the inhaled air stream at the entrance to the extrathoracic region. Alveolar NO concentrations (F_A_NO), NO uptake efficiency (defined as the mass of NO absorbed from the alveolar region divided by the mass of NO inhaled), and exhaled NO concentrations (F_E_NO) were compared between the present model and that proposed by Heinonen *et al.*[11]. In addition, model predictions were compared with exhaled peak and end-tidal NO concentrations measured in pigs by Heinonen *et al.*[11] for the cases of constant 5 ppm NO delivery, and pulsed delivery of 100 nmol NO/min.

Subsequent calculations were performed using the present model to simulate constant and pulsed delivery of NO to adult humans. A base parameter set was defined with FRC of 3000 ml, D_L_NO of 150 ml/min/mm Hg, tidal volume of 500 ml, breath frequency of 12 min^-1^, inspiratory time fraction of 1/3, and expiratory time constant of 0.6 s. Individual parameters were varied to explore their effects on NO uptake and uptake efficiency. The breathing pattern took the form shown in Figure 2, with constant inhalation flow and an exponentially-decaying exhalation flow. The coefficient *A* defining the peak expiratory flow was calculated for a given inhaled tidal volume, expiratory time constant, and expiratory time so as to set the exhaled tidal volume equal to the inhaled tidal volume.

Proportional delivery was modeled using a constant 20 ppm inhaled concentration of NO. Three different pulse durations, *t*_*pulse*,_ were studied: 100, 200, and 400 ms. Pulses were modeled as square waves as depicted in Figure 2. Each pulse delivered 8 μg NO/breath (equivalent to 267 nmol NO/breath). This dose was chosen based on initial calculations performed using the model so as to yield equivalent alveolar NO concentration and uptake for the base parameter set as the constant 20 ppm delivery case. For *t*_*pulse*_ of 100, 200, and 400 ms, 800 ppm NO in balance N_2_ was introduced at 75, 37.5 and 18.75 mL/s, respectively. Again, the NO/N_2_ stream was assumed to be well mixed with the inhaled air stream at the entrance to the extrathoracic region. The effect of delay between the start of inspiration and pulse delivery was studied by setting a pulse delay, *t*_*pulse delay*_, of 0, 50, 100, 200 ms.

## Results

### Model verification

Table 2 lists peak and end tidal values of the alveolar NO concentration, F_A_NO, and exhaled NO concentration, F_E_NO, predicted using the present model and the model presented by Heinonen *et al.*[11], as well as average peak and end tidal values of F_E_NO from experiments in intubated, mechanically ventilated pigs presented by the same authors. NO uptake efficiency as determined by the two models is also presented. In general, very close agreement was seen between the two models.

**Table 2:** Comparison of nitric oxide concentrations and uptake efficiencies in intubated, mechanically ventilated pigs

	**F**_**A**_**NO, peak**	**F**_**A**_**NO, end tidal**	**F**_**E**_**NO, peak**	**F**_**E**_**NO, end tidal**	**Uptake**
**Delivery mode**	**[ppb]**	**[ppb]**	**[ppb]**	**[ppb]**	**Efficiency**
*Present model:*					
5 ppm constant	1066	216	5000	840	0.49
100 nmol/min pulsed	100	17	79	68	0.80
*Heinonen et al.*[11]*model:*					
5 ppm constant	1250	250	5000	820	0.51
100 nmol/min pulsed	125	25	90	67	0.83
*Heinonen et al. *[11]data*:					
5 ppm constant	---	---	4700	890	---
100 nmol/min pulsed	---	---	90	60	---

*As reported in Heinonen et al.[11]*based on experimental regression values.*

*F*_*A*_*NO = Alveolar fraction of NO.*

*F*_*E*_*NO = Exhaled fraction of NO.*

Uptake Efficiency = (NO uptake)/(NO inhaled).

### Constant concentration versus pulsed dosing

**Figure 3:**
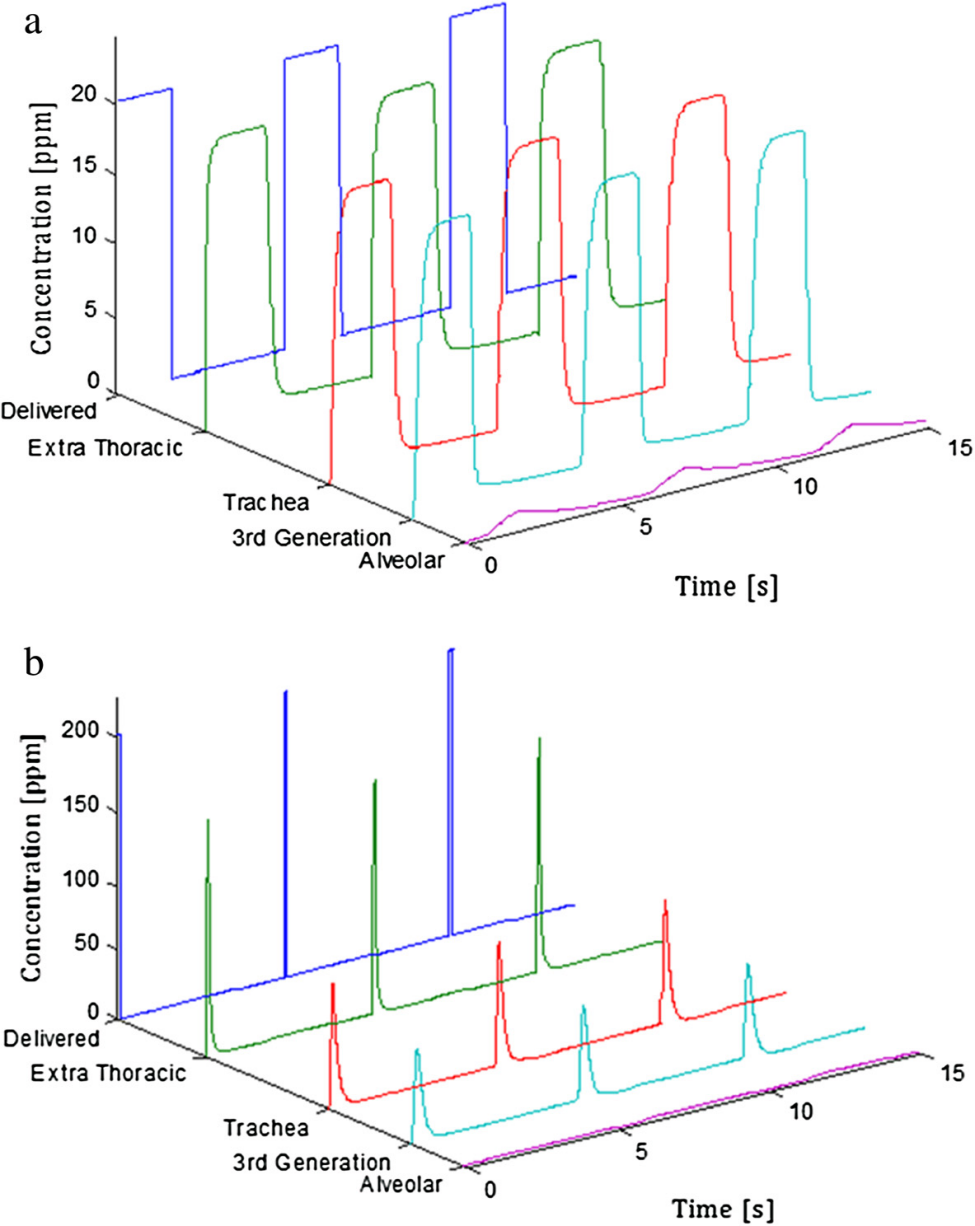
**Regional variation of NO concentration over time.** Shown for **(a)** delivery of a constant 20 ppm concentration during inhalation and **(b)** delivery of a 100 ms pulse containing 800 ppm NO at 75 ml/s into the inhalation flow, sequenced with the start of each inhalation with zero pulse delay. Note the difference in concentration scales between the two plots. Alveolar concentrations are displayed on a more appropriate scale in Figure 4.

**Figure 4:**
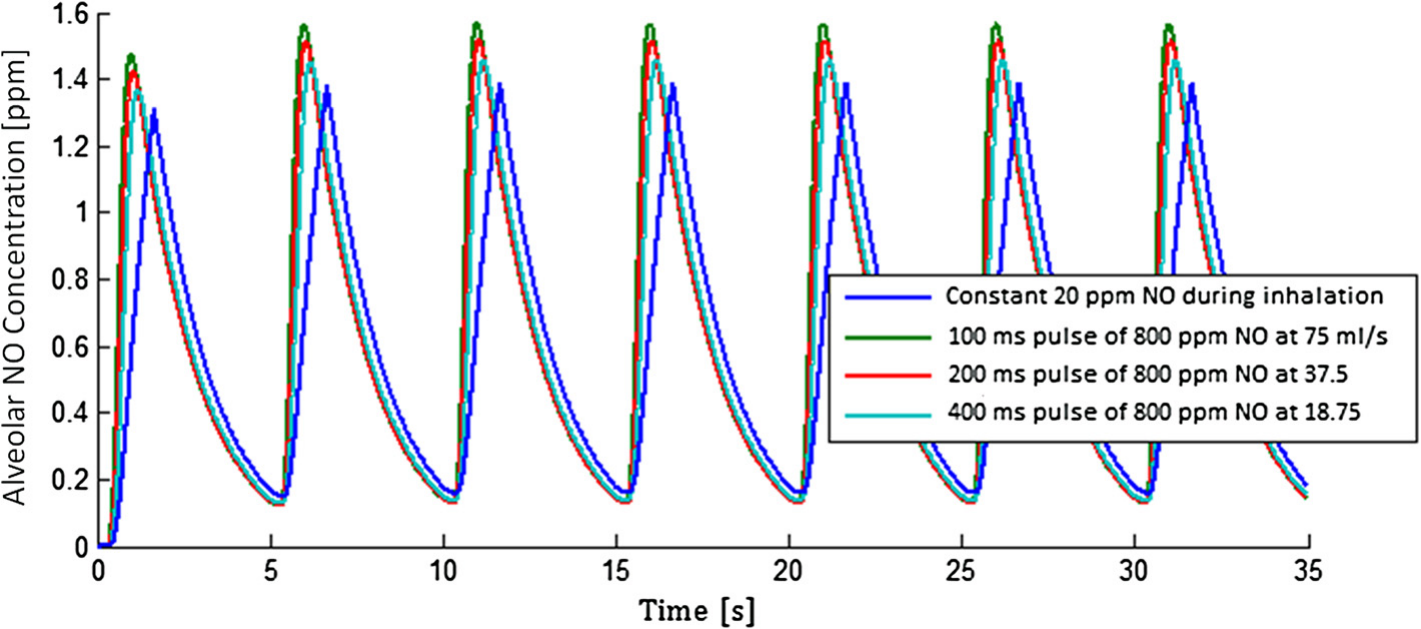
**Alveolar NO concentration (F**_**A**_**NO) versus time for the first 7 breaths after starting constant or pulsed NO delivery.** Pulsed dosing was sequenced with the start of each inhalation with zero pulse delay.

Figure 3 displays variation of NO concentration over time and location from delivery through the segmental bronchi (generation 3), and in the alveolar region, for constant 20 ppm delivery, and for delivery of a 100 ms pulse containing 800 ppm NO at 75 ml/s into the inhalation flow, with zero pulse delay. Concentrations for both delivery modes are presented for the base parameter set. Figure 4 shows alveolar concentrations at a more appropriate concentration scale, for both the constant 20 ppm delivery, and for the three pulse delivery cases, again for the base parameter set. The pulse volumes were set so as to achieve very similar alveolar concentrations as for the constant 20 ppm case.

**Figure 5:**
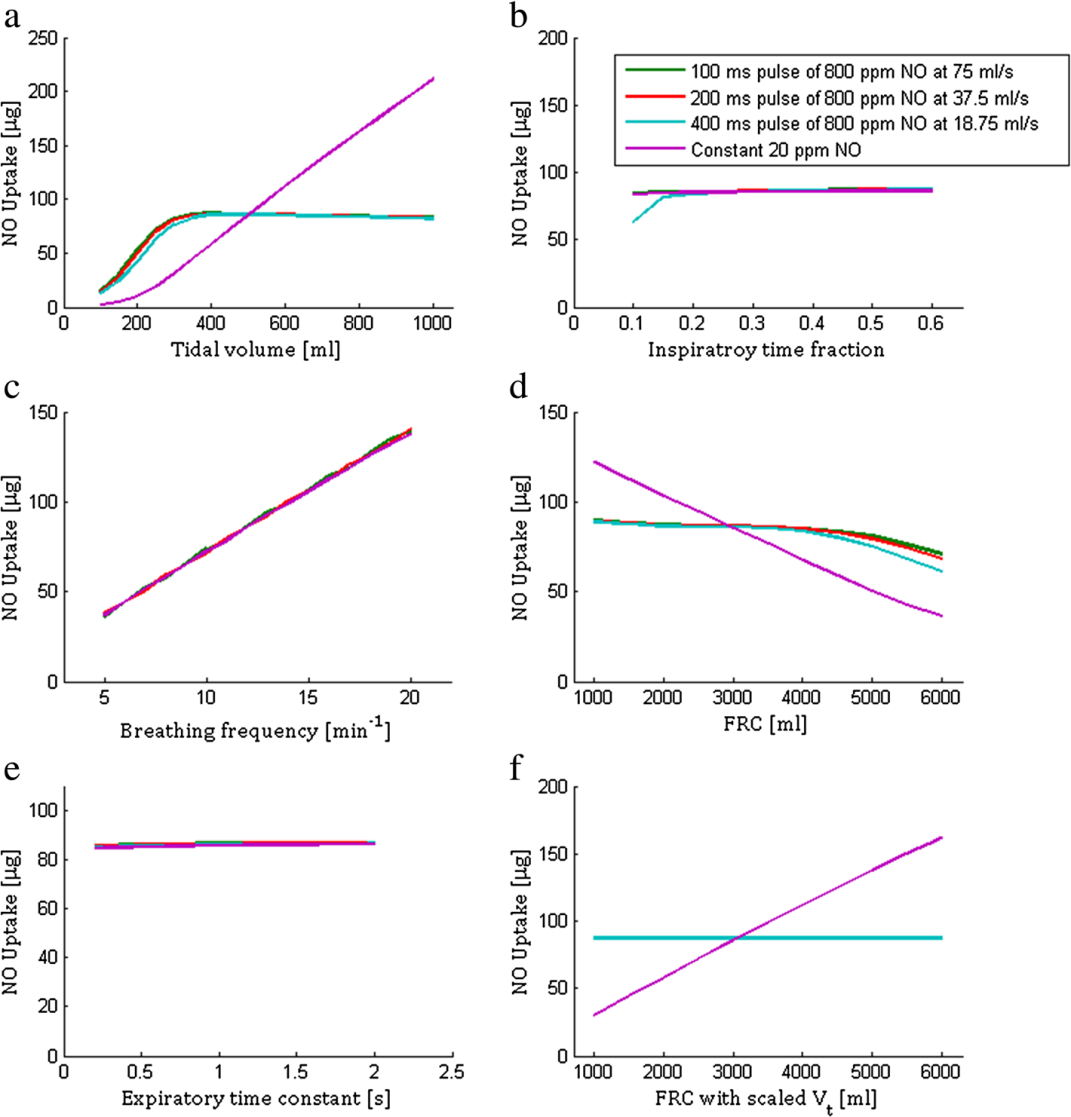
**Total NO uptake over a 60 seconds time period for pulsed and constant concentration delivery.** Plotted with varying **(a)** tidal volume, **(b)** inspiratory time fraction, **(c)** breathing frequency, **(d)** functional residual capacity (FRC), **(e)** expiratory time constant, and **(f)** varying FRC together with tidal volume, where tidal volume is scaled in proportion to FRC. When not the varied parameter, the following values were used: FRC = 3000 ml, D_L_NO = 150 ml/min/mm Hg, tidal volume = 500 ml, breath frequency = 12 min^-1^, inspiratory time fraction = 1/3, and expiratory time constant = 0.6 s.

**Figure 6:**
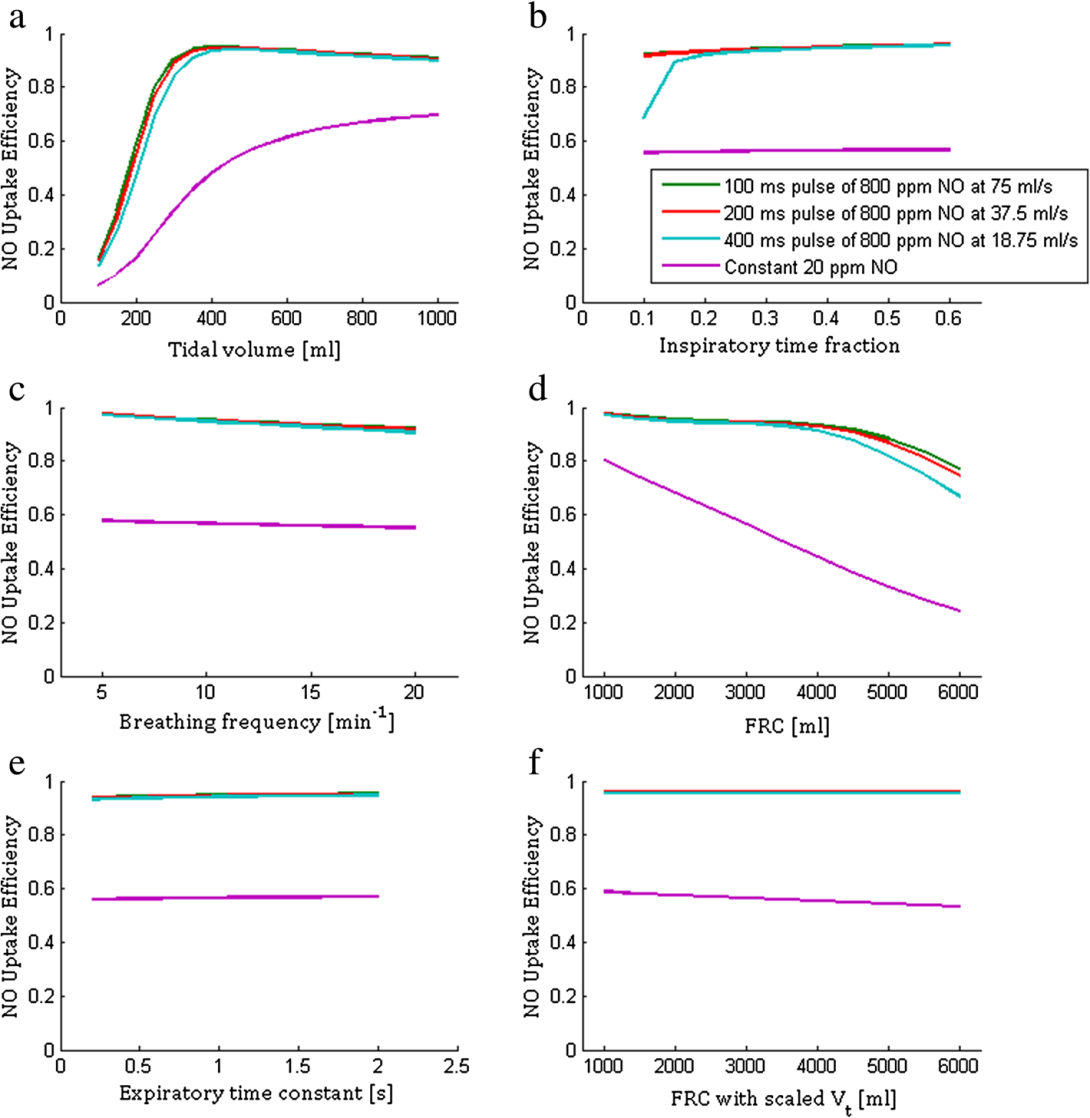
**NO uptake efficiency for pulsed and constant concentration delivery.** Plotted with varying **(a)** tidal volume, **(b)** inspiratory time fraction, **(c)** breathing frequency, **(d)** functional residual capacity (FRC), **(e)** expiratory time constant, and **(f)** varying FRC together with tidal volume, where tidal volume is scaled in proportion to FRC**.** When not the varied parameter, the following values were used: FRC = 3000 ml, D_L_NO = 150 ml/min/mm Hg, tidal volume = 500 ml, breath frequency = 12 min^-1^, inspiratory time fraction = 1/3, and expiratory time constant = 0.6 s.

Variation in NO uptake and NO uptake efficiency with breathing and physiological parameters is shown in Figures 5 and 6, respectively. All plots in Figure 5 include a point of intersection between the pulsed and constant delivery modes, consistent with the choice of pulse volume to yield equivalent alveolar NO concentration as constant 20 ppm delivery for the base parameter set. No points of intersection between modes exist in Figure 6, where in all cases the NO uptake efficiency is significantly lower for constant than for pulsed delivery.

### Effects of pulse delay

Uptake efficiency is provided is Table 3 for varying time delays between the start of inhalation and delivery of the NO pulse. Data is provided for the base parameter set, with tidal volume (V_t_) of 500 ml and breathing frequency (f) of 12 min^-1^, as well as for three other combinations with V_t_ = 250 ml and/or f = 25 min^-1^. All other parameters were held constant at values defined for the base parameter set. While uptake efficiency remained above 0.9 for all combinations of pulse time and delay for V_t_ = 500 ml and f = 12 min^-1^, efficiency decreased dramatically with pulse time and delay for the case of rapid, shallow breathing with V_t_ = 250 ml and f = 25 min^-1^.

**Table 3:** Effects of pulse time and pulse delay on nitric oxide uptake efficiency for varied breathing patterns

		**Uptake efficiency**			
**Pulse**	**Pulse**	**Vt = 500 ml**	**Vt = 500 ml**	**Vt = 250 ml**	**Vt = 250 ml**
**Time [ms]**	**Delay [ms]**	**f = 12 min**^**-1**^	**f = 25 min**^**-1**^	**f = 12 min**^**-1**^	**f = 25 min**^**-1**^
100	0	0.94	0.89	0.88	0.81
	50	0.94	0.89	0.85	0.72
	100	0.94	0.89	0.81	0.59
	200	0.94	0.88	0.70	0.24
200	0	0.94	0.89	0.84	0.70
	50	0.94	0.89	0.80	0.57
	100	0.94	0.88	0.76	0.42
	200	0.94	0.86	0.63	0.14
400	0	0.94	0.88	0.74	0.42
	50	0.94	0.86	0.68	0.31
	100	0.94	0.81	0.61	0.22
	200	0.93	0.62	0.46	0.07

### Uptake efficiency versus D_L_NO

**Figure 7:**
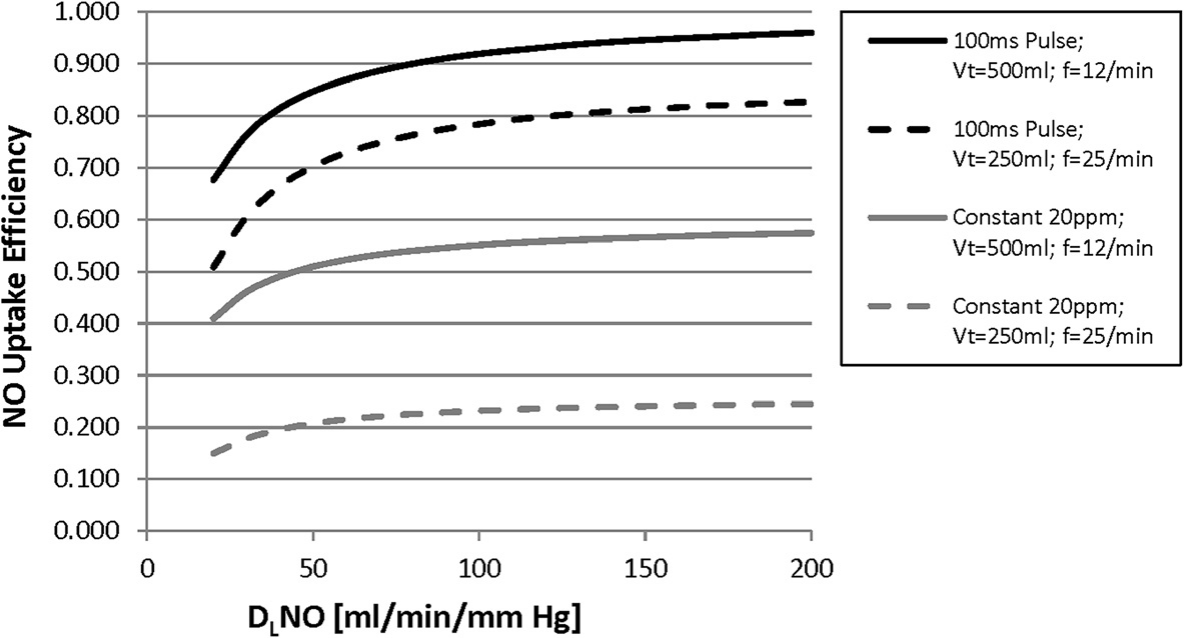
**NO uptake efficiency is plotted against D**_**L**_**NO for pulsed and constant concentration delivery for two breathing patterns.** FRC was held constant at 3000 ml, as was inspiratory time fraction at 1/3.

Figure 7 plots variation in uptake efficiency with D_L_NO for constant delivery of 20 ppm NO and for the 100 ms pulsed delivery, for two breathing patterns. In all cases studied, uptake efficiency was relatively constant for D_L_NO larger than ~80 ml/min/mm Hg, increasing by less than 0.1 as D_L_NO increased up to 200 ml/min/mm Hg. However, uptake efficiency became more sensitive to D_L_NO at values less than ~80 ml/min/mm Hg, and began to drop off sharply as D_L_NO fell below ~50-60 ml/min/mm Hg.

## Discussion

Uptake of NO from the alveolar region depended on pulse timing, tidal volume, respiratory rate, lung and dead space volume, and the diffusing capacity of the lung for NO (D_L_NO). In general, uptake efficiency was consistently higher for pulsed NO delivery than for constant concentration delivery. As will be discussed below, pulse time and delay influenced uptake efficiency more significantly as breath frequency increased, and as tidal volume decreased. In these circumstances, gas mixing in the conducting airways played an important role in determining uptake efficiency for pulsed delivery.

### Model verification

In order to verify calculations made using the model presented above, predictions of alveolar and exhaled NO concentrations, and NO uptake efficiency, were compared with those made previously by Heinonen *et al.*[11]. Those authors constructed a model simulating bulk convection through a single channel, representing the conducting airways, supplying an elastic compartment, representing the alveolar region, and found good agreement with peak and end-tidal exhaled NO concentration measured during pulsed and constant concentration NO delivery to intubated, mechanically ventilated pigs. As seen in Table 2, predictions made using the present model were in close agreement with those made by Heinonen *et al.*[11]. The agreement between these two distinct models, and their consistency with *in vivo* data from an animal model, provides a measure of confidence in moving forward to make predictions regarding NO delivery to humans.

### Constant concentration versus pulsed dosing

One practical application of the model presented here is to guide the design of pulsed NO dosing systems. A logical starting point is to tune pulse volume and timing so as to produce equivalent alveolar concentration and uptake as predicted for delivery of a given constant NO concentration, where dose–response relationships are more established. This was the approach adopted in selecting the 8 μg NO/breath pulses studied herein. As shown in Figure 4, for the base set of input parameters, variation in alveolar concentration over time is very similar for the three pulsed delivery modes as for constant delivery of 20 ppm inhaled NO. NO uptake was also nearly identical between the pulsed and constant modes. Each input parameter was then varied independently to assess its influence on NO uptake, and results are presented in Figure 5. The same calculations are presented in terms of uptake efficiency in Figure 6. With the exception of respiratory rate, variation of NO uptake is predicted to be less influenced by input parameter variation for pulsed than for constant dosing. In particular, NO uptake for pulsed delivery is predicted to be relatively insensitive to tidal volume and FRC, except when tidal volume becomes small or FRC large, in which cases uptake is seen to decrease. Noting that conducting airway volume scaled with FRC, the latter effect arises due to incomplete transport of NO through the conducting airways during inhalation, such that an increasing fraction of NO is exhaled. When tidal volume was allowed to increase in proportion with FRC, as in Figures 5f and 6f, NO uptake and uptake efficiency remained constant for pulsed dosing as FRC varied.

Of note, uptake efficiency was predicted to remain significantly greater for pulsed dosing than for constant dosing in all cases presented in Figure 6. This is seen as an advantage of pulsed delivery systems, in that less NO may be required to maintain an equivalent therapeutic effect. A potential further advantage of pulsed dosing systems is to improve correlation between NO dose and patient response. Under the assumption that therapeutic effects are closely related to uptake, uptake efficiency would ideally remain near 1.0, or at least remain constant, such that the rate of NO uptake could be closely estimated based on the rate of NO delivery. This would allow dosing to be performed through monitoring the absolute amount (mass, volume, moles) of NO delivered, with the potential to improve dose–response correlations [10,11].

### Effects of pulse delay

While uptake efficiency remained near 1.0 for the majority of the parameter range presented in Figure 6, the case of low tidal volume was further examined, as reported in Table 3. Here, the delay time between onset of inhalation and pulse delivery was also considered. At a resting breathing pattern with tidal volume of 500 ml and breathing frequency of 12 min^-1^, uptake efficiency remained above 0.9 even for the extreme case of a 400 ms pulse, with 200 ms delay. In contrast, for rapid, shallow breathing with tidal volume of 250 ml and breathing frequency of 25 min^-1^, uptake efficiency was lower, and much more sensitive to both pulse time and delay. In order to minimize these effects, a pulse time of 100 ms or less, with delay less than 50 ms, is desirable. Making a comparison with pulsed oxygen delivery devices, as have previously been adapted for ambulatory NO delivery through nasal cannulae [12,13], a 50 ms delay is within the range of values reported for commercially available devices [23]. In turn, a pulse time of 100 ms is near reported values for the lowest pulse setting on the majority of devices [23]. Given that required pulse volumes for high concentration NO source gas will generally be smaller than for oxygen, pulse times at or below 100 ms are technically feasible.

### Uptake efficiency versus D_L_NO

All results discussed above were obtained from calculations in which the D_L_NO was fixed at 150 ml/min/mm Hg. This falls approximately in the middle of the range reported by Zavorsky *et al.*[24] for healthy adults performing single-breath-hold maneuvers at rest. Significant variation between subjects is expected even in healthy populations, and certainly in patients with respiratory or cardiovascular disease. For example, in patients with pulmonary arterial hypertension (PAH), average D_L_NO values have been reported as approximately 60–80 ml/min/mm Hg [25,26], with values below 50 ml/min/mm Hg reported in individual patients [25]. Figure 7 demonstrates considerable sensitivity of uptake efficiency to D_L_NO within lower ranges, especially for pulsed delivery. In contrast, at higher ranges, uptake efficiency was much less sensitive to D_L_NO, indicative of excess capacity to take up NO at typical therapeutic dose levels in lungs which maintain normal, or near-normal, alveolar membrane conductance. It is interesting to comment that even for the most efficient case studied, 100 ms pulse with zero delay, uptake efficiency is predicted to lie between approximately 0.7 and 0.9 for D_L_NO values typical of PAH patients. This presents an apparent discrepancy with clinical experience delivering pulsed NO to PAH patients, where no detectable exhaled NO has been reported [13,15], suggesting an uptake efficiency near 1.0. It may be that exhaled NO concentrations in clinical evaluations were simply below the detection limit of the analyzers employed (peak exhaled NO would likely not exceed 1 or 2 ppm, and last fractions of a second, while end-tidal values would lie well below 1 ppm).

Based on present understanding, it appears that the amount of NO delivered is not directly predictive of the amount of NO uptake, such that monitoring NO delivery alone cannot be considered a substitute for monitoring uptake. As variation in uptake efficiency is more pronounced at low D_L_NO, considerable dose titration may be required for patients with initially low D_L_NO that receive NO therapy. This is true for both pulsed dosing and constant concentration dosing. In relative terms, pulsed dosing offered considerable improvement in uptake efficiency compared with constant concentration dosing even for the lowest D_L_NO values studied.

### Nitrogen dioxide production

It may be noted that reaction between NO and oxygen to produce nitrogen dioxide (NO_2_) has not been included in the present model description. In fact, a version of the model was built to include such effects, but NO_2_ production was extremely small. This can be deduced from back of the envelope calculations, given that the chemical kinetics and rate of reaction between NO and oxygen are well known [27]. For example, at a representative inhalation flow rate of 300 ml/s, the residence time of gases passing through the conducting airways is less than 1 s. For 20 ppm NO reacting with 100% oxygen, less than 10 parts per *billion* (ppb) NO_2_ are produced per second. While initial NO concentrations are higher for pulsed delivery, dispersion is rapid (Figure 3b) such that these higher concentrations are very short lived. In the alveolar region, NO concentrations are much lower: assuming an alveolar NO concentration maintained at 1 ppm through supply from the conducting airways, the time to produce 1 ppm NO_2_, even in 100% oxygen, is more than 10 hours – and this assumes NO_2_ accumulates in the alveolar region with no uptake. While such conditions may be possible during extended NO delivery to patients with poorly ventilated and poorly perfused lung regions, the risk of NO_2_ production in the lungs is small in comparison with the risk of NO_2_ developing in breathing circuits used to convey gases to the patient. In the latter case, the combination of sufficiently large NO concentrations and residence times to produce appreciable amounts of NO_2_ may exist, and precautions to avoid excess NO_2_ production have been documented [10,28].

### Conducting airways model

**Figure 8:**
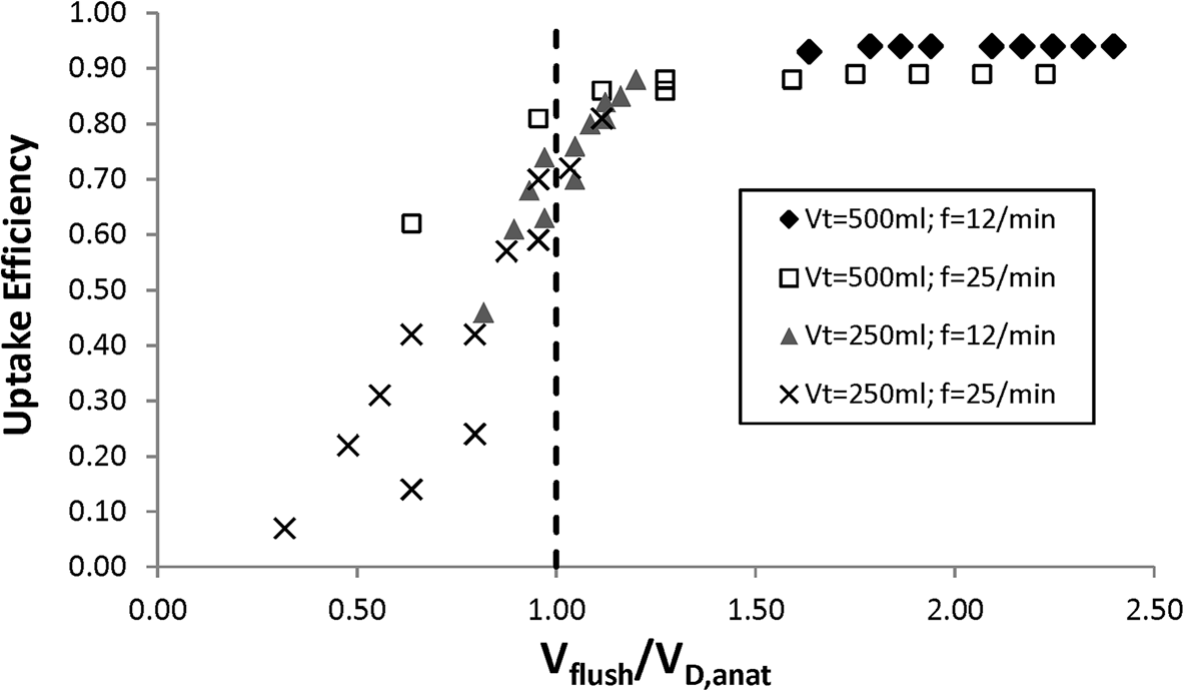
**Uptake efficiency for pulsed delivery from Table**3**is plotted against the ratio between flush volume (V**_**f**_**) and anatomical dead space volume (V**_**D,anat**_**).** The flush volume is defined as the tidal volume minus the total volume inhaled during the delay time and pulse time.

The present model treated the conducting airways as a series of individual generations, and included axial dispersion of NO through the empirical coefficient proposed by Scherer *et al.*[21]. One may reasonably question whether modelling axial dispersion provides any significant insight in comparison with simpler models. In describing pulsed oxygen delivery, other investigators have used idealized bulk analysis based on relative volumes of the conducting airways (anatomical dead space) and tidal breath [29,30]. With regards to the present work, Figure 8 highlights the significance of including mixing in the conducting airways as opposed to a bulk analysis based on relative airway and tidal volumes. The ‘flush volume’, V_f_, is defined as the tidal volume minus the total volume inhaled during the delay time and pulse time. Under a volumes-based analysis, so long as the flush volume is greater than the volume of the anatomical dead space, all NO delivered in the pulse is swept into the alveolar region, and made available for uptake. In contrast, Figure 8 displays uptake efficiency as low as 0.7 in the region of the plot where flush volume exceeds dead space volume, indicating that a portion of NO remains in the conducting airways at the end of inhalation (this was confirmed by viewing generational concentration data). Accordingly, gas mixing in the conducting airways plays an important role in determining the efficiency of pulsed delivery systems.

### Limitations

Several limitations of the present model present opportunities for future work. First, extension to multiple paths and multiple compartments would allow the interplay between regional variation of respiratory mechanics, ventilation, perfusion, and uptake efficiency to be explored. Second, the possibility of significant concentration gradients within the alveolar air spaces could be explored by moving to a multi-generation model of the alveolar region [31,32], with potential impact on total uptake efficiency. Finally, the present model treated the extrathoracic airway as a single tube, representative of an endotracheal tube, while pulsed delivery has frequently been linked with long term administration of NO through nasal cannulae. Depending on the cannula design, mixing of NO source gas with room air, and with any additional supplemental oxygen, will occur during transit through the nasal air passages and will determine boundary conditions entering the trachea. More accurate representation of concentration profiles exiting the extrathoracic airway will likely require bench experiments, or computational fluid dynamics simulations, specific to a given patient interface and nasal airway geometry.

## Conclusions

In conclusion, pulsed NO delivery offers significant improvement in uptake efficiency compared with constant concentration delivery. However, uptake from the alveolar region can depend on pulse timing, tidal volume, respiratory rate, lung and dead space volume, and D_L_NO. It is predicted that variation in uptake efficiency with breathing pattern can be moderated using a pulse time of less than 100 ms, with a delay of less than 50 ms between the onset of inhalation and pulse delivery. Nonlinear variation in uptake efficiency with D_L_NO was predicted, with uptake efficiency falling off sharply as D_L_NO decreased below ~50-60 ml/min/mm Hg. Gas mixing in the conducting airways played an important role in determining uptake efficiency for pulsed delivery.

## Competing interests

At the time of the study, all authors were employed by Air Liquide. Air Liquide markets medical nitric oxide in several geographic regions.

## Authors’ contributions

AM conceived of the study, participated in developing the model, and wrote the manuscript. CJ participated in developing the model, wrote code to conduct the model calculations, and contributed to drafting the manuscript. IK participated in the conception and design of the study, and edited the manuscript. GC participated in the conception and design of the study. All authors read and approved the final manuscript.
